# Multiple events case–control study in a prospective cohort to identify systemic, cellular, and molecular biomarkers of obesity-induced accelerated aging in 30-years-olds: the ObAGE study protocol

**DOI:** 10.1186/s12877-022-03032-4

**Published:** 2022-05-02

**Authors:** P Correa-Burrows, R Burrows, C Albala, FA Court, F Salech, G Sanhueza, C Gonzalez-Billault

**Affiliations:** 1 Institute of Nutrition & Food Technology, Universidad de Chile, Santiago, Chile; 2 Center for Integrative Biology, Universidad Mayor, Santiago, Chile; 3 Geroscience Center for Brain Health and Metabolism (GERO), Santiago, Chile; 4 Buck Institute On Aging Research, Novato, CA USA; 5 Faculty of Medicine, Universidad de Chile, Santiago, Chile; 6 Faculty of Social Sciences, Universidad de Chile, Santiago, Chile; 7 Faculty of Sciences, Universidad de Chile, Santiago, Chile

**Keywords:** Obesity, Aging, Epigentic age, Resilience, Early-onset cardiometabolic risk, Sarcopenia, Dysbiosis

## Abstract

**Background:**

Aging is characterized by a progressive loss of capacities linked to fundamental alterations/damage in multiple cellular and molecular pathways. It is the most significant risk factor for all non-communicable diseases (NCDs). Another contributing factor to the rise in NCDs is obesity. It has been suggested that obesity not only accelerates the onset of metabolic imbalances but also decreases lifespan and impacts cellular and molecular processes in a manner similar to aging. Obesity might accelerate the pace of aging. Guided by a lifecourse approach, we will explore how exposure to obesity in critical developmental stages disrupt homeostatic resilience mechanisms that preserve physiological integrity, inducing an early expression of aging phenotypes. Also, we will determine whether exposure to early psychosocial adversity influences vulnerability to obesity as a risk factor for accelerated aging.

**Methods:**

Multiple events case–control study embedded in a prospective cohort of Chileans at 30-31y, 50% females, of low- to-middle socioeconomic status, who participated in nutrition research since birth. At 23y, 25% had obesity and cardiometabolic risk was high. We will use a multi-layer approach including: anthropometric assessment; DXA scan for body composition; abdominal ultrasound of the liver; stool samples collection and sequencing of the ribosomal RNA 16S gene to characterize the gut microbiome; determination of age-related pro-inflammatory cytokynes and anti-inflammatory miokynes. For the first time in Chile, we will address age-related epigenetic changes using the Horvath´s epigenetic clock. In a subset we will conduct a controlled physical challenge to characterize physical resilience (autophagy).

**Discussion:**

ObAGE is in an excellent position to: approach aging as a process whose expression involves multiple factors from the early stages of a person's life; understand how longitudinal changes in health trajectories impact the biological mechanisms of aging; identify potential resilience mechanisms that help prevent unhealthy aging. Because SLS participants are still young, our research setting combined with advanced scientific techniques may identify individuals or groups at risk of early onset health issues. Results from ObAGE may pave the way to address the contribution of obesity to aging through lifespan from cells to systems and might be instrumental to developing interventions to improve health span in the Chilean population.

**Trial registration:**

The proposed study does not consider any health care intervention on human participants.

## Background

Age is a risk factor for many diseases, and improvements in life expectancy increase the chance of developing age-related diseases. Many normal-weight individuals develop non-communicable diseases (NCDs) at older ages; however, people who are overweight or obese may reach the threshold of disease at a younger age. Epidemiological studies consistently show that obesity reduces health span by increasing the risk of cardiometabolic morbidity and mortality and loss of muscle mass and strength. Atherosclerosis [1, 2], insulin resistance (IR) [3–6], and sarcopenia [7–9] are common in normal human aging; however, obesity at an early age speeds up these aging processes decades before they manifest clinically. Although aging is a natural progression, the expression of an aging phenotype is increasingly seen in younger-age populations due to rising levels of obesity, suggesting that excessive fat accumulation may accelerate the pace of aging.

Studies based on large epidemiological datasets aim to explore the impact of obesity on the aging process at the systems level. However, going from systems to cells and molecules is crucial in understanding the potential role of obesity as an aging risk factor. Omitting the cellular and molecular levels makes it difficult to understand the pathways by which obesity might accelerate aging [10–13], and identify resilience mechanisms that help prevent the early loss of physiological integrity and the development of diseases [14]. It has been suggested that obesity and aging share several physiological traits or hallmarks related to dysfunctional adipose tissue. Obesity causes oxidative stress and chronic inflammation, which negatively influence mitochondrial function, nutrient sensing, intercellular communication, and proteostasis [13, 15, 16]. Obesity promotes the aging process by inducing senescence –a condition in which a cell no longer can proliferate–, and the resulting pro-inflammatory secretory phenotype contributes to obesity-related comorbidities, particularly those with underlying IR (e.g., type-2 diabetes) [17]. Also, obesity appears to accelerate epigenetic changes associated with aging in specific tissues [18, 19]. By sharing a similar spectrum of phenotypes, obesity might speed up the progressive loss of physiological integrity common in aging. Therefore, a thorough characterization of biological, molecular and epigenetic features will contribute to better define life course trajectories that allow separating healthy from unhealthy aging.

Increased levels of social adversity also relate to aging. Social hallmarks of aging include low socioeconomic status, minority status, adverse life events, adverse psychological states, and adverse behaviors [20]. Models of human health support the notion of ‘aging’ as a lifelong process that begins very early in life [21, 22]. Health at older ages is affected by experiences through life, with specific developmental stages being particularly critical. A growing body of literature reports an increased risk of adolescent and adult cardiometabolic disorders among those maltreated in childhood (e.g., neglect, abuse, household dysfunction, exposure to crime, economic disadvantage) [23–25]. The long-term consequences of childhood adversity have been explained through increased vulnerability to succeeding stressful events [26, 27]. This vulnerability may involve persistent dysregulation of stress responses, which is thought to result from exposure to stressors during sensitive developmental periods [28, 29]. Early life adversities may have a lifelong influence on the hypothalamic–pituitary–adrenal (HPA) axis, the primary regulator of the stress response [30, 31]. Acute activation of the HPA axis during stress is necessary for adaptation; however, persistent exposure to elevated levels of stress hormones (e.g., cortisol) results in impaired stress response and susceptibility to metabolic and immune alterations [32, 33].

It follows that a deep understanding of human aging will come from integrating social factors along with biological factors underlying this process into transdisciplinary work. Thus, the Study on Obesity-Induced Accelerated Aging (ObAGE) brings together Geroscience, Public Health, Epidemiology, Clinical Medicine, and Social Sciences to explore how exposure to obesity in crucial developmental stages disrupts homeostatic resilience mechanisms that prevent the early expression of aging phenotypes, and determine whether social position influences vulnerability to obesity as a risk factor for accelerated aging.

## Methods

### ObAGE Study aims

Our overall research goal is to understand how exposure to obesity in critical developmental stages accelerates biological aging and undermines long-term health in a Chilean historical birth cohort of high social vulnerability ang high prevalence of obesity and cardiometabolic risk. Guided by a life-course approach, we propose to evaluate adults in their early thirties and investigate three research aims:

- Research aim 1 – To investigate whether obesity in key developmental stages triggers an early expression of aging at multiple levels (from cells to systems) in adults < 35y. We hypothesize that exposure to obesity in childhood, adolescence, and early adulthood accelerates the expression of an aging phenotype and biological features that speed up the onset of age-related conditions and diseases. Specific aims are to:
  - Explore the role of BMI trajectories throughout the life course as a risk factor to expressing accelerated aging phenotypes.
  - Determine whether obesity in childhood, adolescence, and early adulthood, as defined by BMI and other adiposity measures, accelerates biological age determined by DNA methylation levels.
  - Determine whether obesity in the above-mentioned developmental stages relates to dysbiosis, chronic low-grade inflammation, loss of skeletal muscle mass and strength, and increased progression of cardiometabolic risk to cardiometabolic diseases in young adulthood
- Research aim 2 – To determine whether exposure to early psychosocial adversity (e.g., child abuse and neglect, witnessing family violence, having a parent with untreated mental illness, financial distress) confers greater vulnerability to obesity-induced accelerated aging. The underlying hypothesis states that exposure to psychosocial adversity in infancy, childhood, and adolescence leads to impaired functioning of the HPA-axis and impaired response to stress, increasing vulnerability to obesity-induced accelerated aging. Specific aims are to:
  - Determine whether obese individuals exposed to early psychosocial adversity are at higher risk of obesity-induced accelerated aging than obese individuals not exposed to such stressors.
  - Determine differences in the obesity-induced aging phenotype after controlling for exposure to early psychosocial adversity.
  - Explore the direct and indirect contribution of different types of psychosocial adversities in infancy, childhood, and adolescence to obesity-induced accelerated aging.
- Research aim 3 – To investigate the autophagy response in peripheral blood mononuclear cells (PBMCs) following an acute bout of high-intensity interval training (HIIT) with treadmill running in obese vs. non-obese participants. We hypothesize that HIIT will stimulate autophagy in PBMCs in both groups; however, this response will differ after controlling for obesity status. Specific aims are to:
  - Investigate weight-status differences in basal and stress-induced autophagy in PBMCs.
  - Investigate weight-status differences in protein expression of LC3I, LC3II, and p62 in response to acute exercise in adults < 35y.

### Setting

**Fig. 1:**
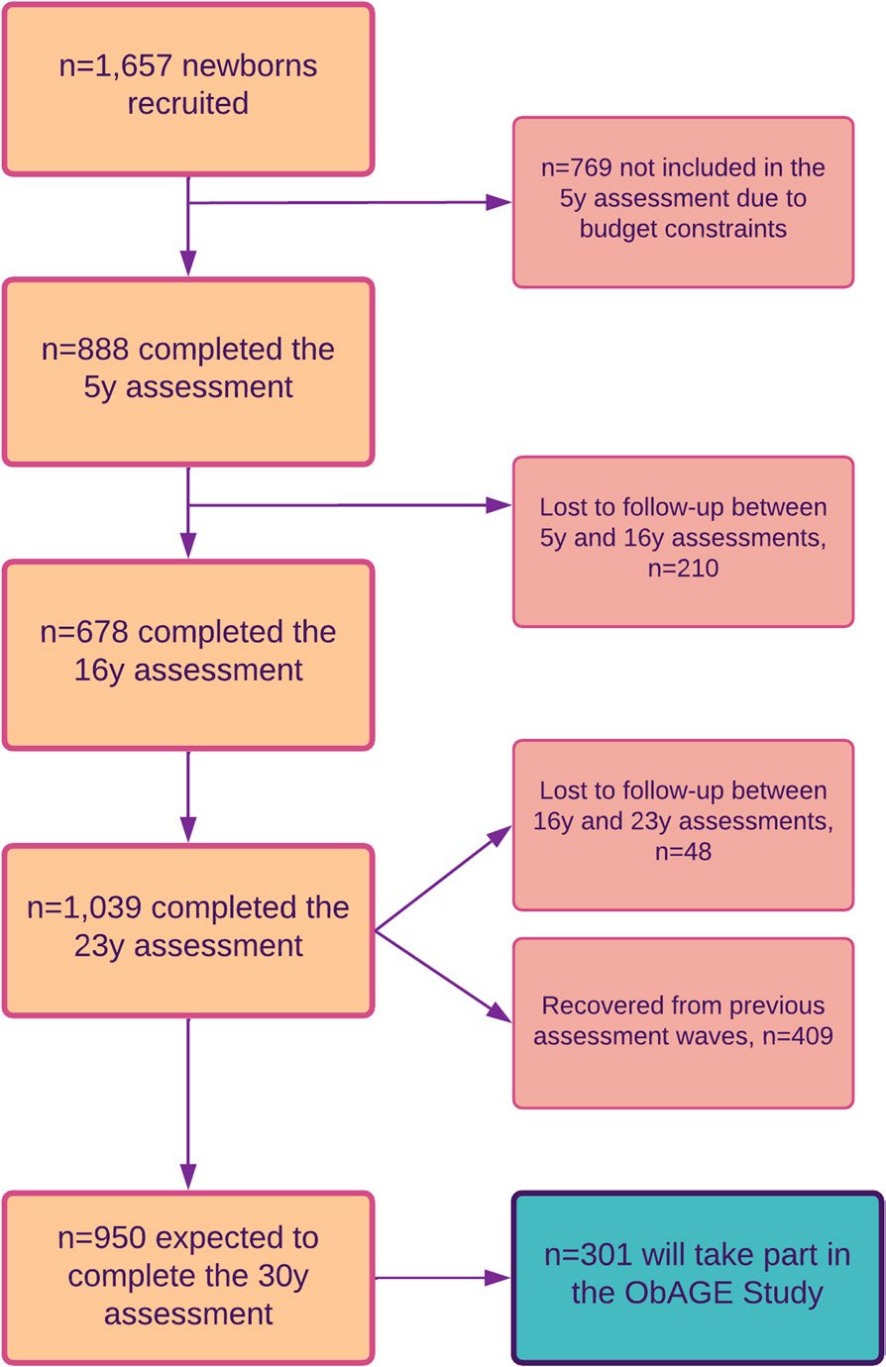
The Santiago longitudinal study, 1992–2022

**Fig. 2:**
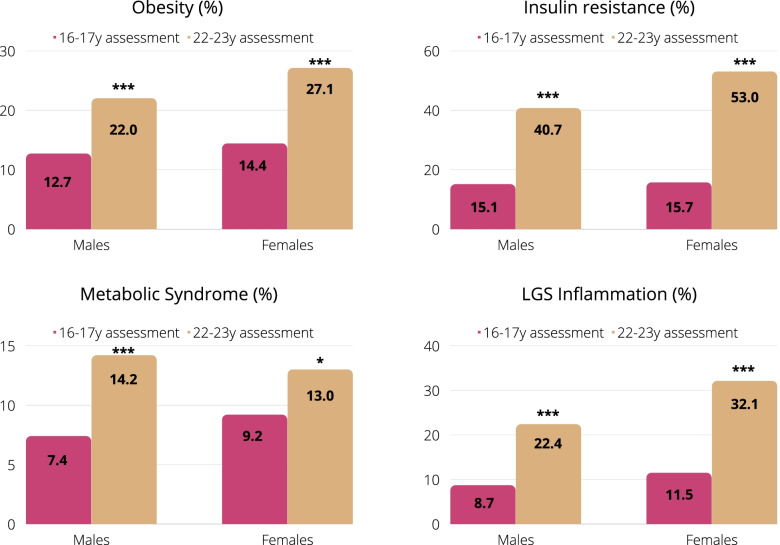
Obesity and cardiometabolic risk from adolescence to emerging adulthood in the Santiago Longitudinal Study (*n* = 630). The subset includes participants with repeated measures at 16y and 23y. Exact McNemar’s test: *Significant at *P* ≤ 0.05. **Significant at *P* ≤ 0.01. ***Significant at *P* ≤ 0.001. Obesity defined as BMI ≥ 30 kg/m^2^. Insulin Resistance defined as HOMA-IR > 2.6. Metabolic Syndrome defined according to the AHA/IDF joint criteria. LGS Inflammation defined as hs-C reactive protein ≥ 3 mg/L according to CDC/AHA

We will capitalize on a prospective birth cohort (the Santiago Longitudinal Study, SLS) of ≈1000 Chileans at 30-31y, 50% females, of low- to middle SES, who participated in research related to nutrition and development as infants with follow-up at 1y, 5y, 10y, 12y, 14y, 16y, 21y, and 23y (see Fig. 1). They were born in the first half of the 1990s, at term, of uncomplicated vaginal births, normal weight (> 3.0 kg), and free of chronic health problems [34]. Recruitment occurred during a dramatic epidemiological transition associated with decreasing undernutrition and increasing NCD [35]. In 2009, when the SLS participants were in their adolescent years, a study of 'Biopsychosocial determinants of adolescent obesity and cardiovascular risk’ initiated in this setting.' Assessment of anthropometric and cardiometabolic markers was repeated in early adulthood (23y), and a 3^rd^ wave started in late 2021. Field-work for the 23y wave was conducted in 2015–2018. A total of *n* = 1,039 participants completed the assessment. While SLS participants were born healthy and well-nourished children, their parents or grandparents were exposed to undernutrition, which may result in a higher risk of cardiometabolic alterations in offspring (thrifty phenotype). At 23y, we found that 25% had obesity, 14% had metabolic syndrome, 47% had IR, 28% had non-alcoholic fatty liver, and 27% had low-grade systemic inflammation [9, 36] (see Fig. 2). Also, in these participants, a poorer infant psychosocial environment was associated with a higher BMI, blood pressure, triglycerides, total cholesterol, and high-sensitivity C-reactive protein, and a greater prevalence of IR and MetS in adolescence and early adulthood [37, 38].

### Design

Multiple events case–control (MECC) study embedded in a prospective cohort. In the MECC design, there is a known and enumerated cohort, from which subjects are selected for additional measurements. The MECC design is superior to case-cohort or nested case–control studies as the former eliminates bias and improves the efficiency of the analysis of the same data [39]. To be eligible SLS participants must have complete data in all assessments waves, and fall into one of the following categories of lifetime BMI trajectory: (1) participants always having a BMI in the healthy range (never-OB); (2) participants with obesity starting in adolescence and remained obese into adulthood (recent onset-OBs); (3) participants who were obese in early childhood and remained obese into adulthood (persistent-OBs) (see Supplementary Material). A sample size of *n* = 300 allows testing the study hypotheses and detecting small differences (*f* = 0.18), at α = 0.05 and 1-β = 0.8, in a 1:1:1 design.

### Measurements and procedures

A 3-h clinical assessment is planned. Participants will attend the evaluation early in the morning (8.00 AM) in a fasting state (8–10 h). They will undergo anthropometric and body composition assessments, liver ultrasound, and will provide a 25 ml venous blood sample to measure inflammatory, nutrient sensing, muscle/bone-derived, aging-dependent and liver-related markers. Additionally, they will receive a kit to provide a stool sample no later than seven days after the clinical assessment to analyze their gut microbiome. A subset (*n* = 60) will undergo a physical fitness challenge.

#### Measurements & procedures (Aims 1–2)

##### Anthropometric assessment

Research staff will use standardized procedures to measure weight to the nearest 0.1 kg, using a precision electronic scale (Seca 703, Seca GmbH & co. Hamburg, Germany) and height to the nearest 0.1 cm, using a Holtain stadiometer with the head in the Frankfurt position. Waist circumference (WC) will be measured with a non-elastic flexible tape at the midpoint between the edge of the last rib and the iliac crest and recorded to 0.1 cm (Seca 201, Seca GmbH & co. Hamburg, Germany). As described in previous work [36], measurements will be taken in duplicate (the mean will be used for analysis); if the difference between the two measures exceeds 0.2 kg in weight, and 0.5 cm in height and WC, a third measurement will be mandatory. Mean Body-Mass Index will be estimated.

##### Body composition

As BMI only considers weight and not body composition, the use of BMI is limited. Percentage body fat, muscle mass index, waist-to-height ratio, and waist-to-hip ratio have been shown to better diagnose obesity and assess and predict total and cardiometabolic health, as they provide better information about body composition and fat distribution [40, 41]. SLS participants underwent DXA-based body composition at 16y and 23y. A third assessment is considered for the ObAGE Study. This will allow: (i) relating biological age with more accurate predictors of cardiometabolic risk; (ii) exploring these relations longitudinally; (iii) exploring options for data inter and extrapolation once we have a third measurement. A dual-energy X-ray absorptiometry (DXA) scan (Lunar Prodigy Corp., Madison, General Electric, WI, USA, and software Lunar iDXA ENCORE 2011, Version 13.60.033 © 1998–2010) for body composition will be performed in the fasting state. Body composition will be measured to determine total and percent body fat mass as well as total and percent lean mass in the total body, arms, legs, and trunk. The same procedure will allow determination of bone mineral density and bone mineral content in the total body and specific regions. With a third measurement of these bone health biomarkers at 29-30y, we will be covering the overall period of bone-mass gain in humans.

##### Assessment of the liver

The liver is a key determinant of metabolic abnormalities. NAFLD occurs more often in males than in females and affects mainly the middle-aged and the elderly. At 23y, however, 28% of SLS participants had NAFLD, with no sex differences [42]. An abdominal ultrasound will be performed in SLS participants using a General Electric LogiQ ultra-sonographer with a 4C RS convex multifrequency probe (2–5.5 MHz) (GE Healthcare Systems, Wauwatosa, WI, USA). As described in previous work from our group [42], all examinations will be done by the same operator, and images will be analyzed by two independent observers. Observers will be gastroenterologists with training in abdominal ultrasound interpretation, and will score liver brightness (0–3), diaphragm attenuation (0–2) and vessel blurring (0–1), according to Hamaguchi et al [43]. The maximum score possible is six; values ≥ 4 have good sensitivity (82%) and specificity (84%) for NAFLD diagnosis [42]. Alanine and aspartate aminotransferase will be determined by enzymatic methods (ADVIA 1650, Siemens, Tarrytown, NY). Radioimmunoassay (RIA) techniques will be used for determination of hepatitis B surface antigen and hepatitis C virus antibodies.

##### Microbiome

In the last decade, the microbiome composition has emerged as a crucial component to explain organisms’ selective vulnerability to the deleterious consequences of aging and predisposition to the onset of age-related diseases. It is unclear whether the association between aging and microbiome depends on a decrease in microbial diversity or the loss of certain specific and beneficial microorganisms. Therefore, we aim to explore the relationship between obesity, microbiome, and aging. We expect to understand the composition, functionality, and molecular interactions between the microorganisms involved in the regulation and development of pathologies associated with obesity, and that could regulate the immune system and immunosenescence in the SLS cohort. Stool samples from SLS participants will be collected using standardized techniques and processed in our laboratories. Ribosomal RNA 16S gene will be sequenced using Illumina (MySeq System, Illumina Inc., San Diego, CA), adding the microbiome data to each participant´s file record. We will use these data to characterize the gut microbiome composition, and perform associations between microbiome parameters (including diversity, abundance and specific bacterial groups present in each individual) and the other measurements obtained in this study.

##### Inflammatory biomarkers

Blood samples collected from the cohort will be centrifuged to separate plasma that will be stored in 250 µL aliquots at -80ºC. Most of the biomarkers determination will be done using the Luminex system (Luminex® 100/200™ System with xPONENT® 4.3) that allows the analysis of multiples proteins using fluorescent and infrared beads that can be excited using a laser beam. Each beam is conjugated with a specific antibody designed against the protein of interest. Differences observed in plasma concentrations for each protein will be evaluated for their significance using Mann–Whitney or Kruskal Wallis tests. The following biological markers will be evaluated: IGF-1, IGF-2, OSM, myostatin, Fgf21, Gdf15, Gdf11, Apelin, Musclin, Sparc, Irisin, IL-2, IL-6, IL-10, tumor necrosis factor-α (TNFα), C-reactive protein (CRP), and serum amyloid P component (SAP). To avoid abnormally high levels of inflammatory markers (denoting acute inflammation), participants being ill (e.g., viral infections) at least ten days before the evaluation will have their appointment rescheduled.

##### Epigenetic clock

One of the most recent approaches used to assess the biological age is the utilization of epigenetic mechanisms, namely, DNA methylation. This approach is based on the observations that aging relates to changes in the DNA methylation levels. Horvath’s epigenetic clock allows the determination of the biological age using the Horvath algorithm based on the methylation level of 353 CpG sites. This algorithm yields an estimate of the individual’s DNA methylation age that generally correlates well with a healthy individual’s calendar age and is claimed to be a successful predictor in most cell types and tissues [44]. The acceleration of epigenetic age can be assessed by calculating the deviation of calendar age from epigenetic age (ΔAGE). The evaluation of the epigenetic clock will be done in collaboration with the Epigenetic Clock Development Foundation (Torrance, CA). Illumina DNA sequencing (27 K or 450 k platforms) will be performed and bioinformatics analysis will be conducted with the Epigenetic Clock Software available at Horvath.genetics.ucla.edu.

#### Measurements & procedures (Aim 3)

Because of the young age of the SLS participants, we propose to assess physical resilience using a strenuous physical activity challenge. Considering the changes in the regulation of autophagy in people with obesity [45] and the described effect of acute physical activity on the regulation of autophagy in peripheral blood mononuclear cells (PBMC) in control subjects [46], we will look for changes in the response under physical stress of several essential regulatory proteins of autophagy in PBMC following an acute bout of high-intensity interval training (HIIT) with treadmill running. Thirty obese participants and 30 paired normal-weight controls will be randomly selected from cohort to undergo a physical resilience challenge. After passing a safety assessment for stress tests in patients at risk of coronary heart disease, they will perform a bout of High-Intensity Interval Training (HIIT; 12 bouts for 1 min at 100% Vmax and 1 min at 3 mph) in an 8 h fasting state, following the protocol by Escobar et al [46]. Heart rate, blood pressure, and oxygen saturation will be measured during the procedure. PBMC will be collected (Ficoll® gradient) pre- and 2 h post-exercise. We will measure autophagy by assessing differences in protein expression of LC3I, LC3II, and p62 via western blot analysis. A sample of *n* = 60 in a 1:1 design allows detecting moderate differences between groups at α = 0.05 and 1-β = 0.82.

### Data from previous or ongoing SLS studies

We have available BMI, SES status, and several measurements of individual risk from infancy to 23y. Cardiometabolic biomarkers are available since adolescence. Cortisol will be assessed simultaneously as part of an ongoing neuroendocrine project in the same cohort (see Table 1).

**Table 1:** Available Data in the Santiago Longitudinal Study (29y assessment is ongoing, aiming to relate exposure to early psychosocial adversity to cardiometabolic risk in adulthood)

	Type of Measure	Method/instrument	Infancy assessment (enrollment to 1y)	Childhood Assessment (5,10,12y)	Adolescence assessment (16-17y)	23y assessment	29y assessment
Epidemiology	Demographics	Age, sex, residence area	X	X	X	X	X
	Socioeconomic	Income, economic responsibilities, financial access, human & social capital, future income expectations & worries, SES change.	X	X	X	X	X
	Individual risk (I)	Behavior problems, mental health, emotional regulation, stress, dating violence.	X	X	X	X	X
	Individual risk (II)	child abuse and neglect, intrafamily violence, having a parent with untreated mental illness, financial distress, feeling unsecure	X	X	X	X	X
	Individual risk (III)	Tobacco, alcohol, drug use.			X	X	X
	Individual functioning	Work, relationship, living independently, social network, life events.				X	X
	Education	Academic record, high school completion, college exams, higher education			X	X	X
	Medical history	Acute, chronic, and mental disorders. Records of current and past medication.			X	X	X
	Family medical history (I)	T2D, stroke, myocardial infarction, HTA, dyslipidemia			X	X	X
	Family medical history (II)	Dementia/Alzheimer’s					X
	Diet (I)	Breastfeeding (total and exclusive)	X				
	Diet (II)	Food-frequency data about food and beverages, 24H recall			X	X	X
	Physical activity (I)	Exercise, active commuting, sedentary behavior			X	X	X
	Physical activity (II)	Actigraphic recording			X	X	
	Sleep	Pittsburgh Sleep Quality Index, Munich Chronotype Questionnaire, Epworth Sleepiness Scale			X	X	
Clinical	Anthropometry (I)	Height, weight, BMI, BMI (Z score)	X	X	X	X	X
	Anthropometry (II)	Waist circumference, WtHR, WHR			X	X	X
	Anthropometry (III)	Fat mass, fat-free mass, fat distribution (DXA)			X	X	
	Cardiometabolic (I)	Blood pressure			X	X	X
	Cardiometabolic (II)	Carotid intima thickness, pulse wave velocity.				X	X
	Bone density	BMD, BMDz, BMD, T score, BMC (DXA)			X	X	
Laboratory	Hematology	Full blood count, hematocrit, hemoglobin, platelets, white cell differentials	X	X	X	X	
	Liver function	Liver ultrasound, serum AST, ALT, HCV antibody and HBsAg				X	
	Lipid profile	Total Chol, HDL-Chol, TG, LDL, VLDL			X	X	X
	Glucose control	Glucose, insulin, HOMA-IR, HOMA-β, HOMA-S, Disposition Index			X	X	X
	Inflammatory markers (I)	hs-CRP			X	X	X
	Inflammatory markers (II)	TNFα			X		
	Appetite hormones	Leptin, ghrelin, adiponectin, orexin A			X	X	X
Other	Stress hormones	Cortisol (blood, saliva and hair)					X
	Bio specimens	Extracted DNA				X	
	Bio specimens	Blood, biofluids and derivatives			X	X	X
	Bio specimens	PBMC					X

### Data analysis

For cross-sectional analysis, we will use models based on the law of total variance, such as one-way and two-way ANOVA and their non-parametric equivalents. With some variables, we may use ANOVA for repeated measures. To account for the effect of covariates (to reduce the residual variance and type-2 error and improve statistical power) we will use analysis of covariance, which blends ANOVA and regression. For longitudinal modeling, we will use statistical models based on the likelihood principle, such as the mixed-effects model or hierarchical models and path analysis. Path analysis represents a methodological improvement regarding multivariate techniques used in modeling some health-related issues, particularly the role of social determinants. It allows investigation of more complex models, providing information that could have been previously overlooked, such as how the interrelations among independent variables in a model affect the dependent ones. Hence, it may be particularly useful to explore the connections of social and biological hallmarks of aging. Machine learning will be used for microbiome data.

#### Modelling of BMI trajectories

In the past, we obtained life course BMI trajectories for all participants, despite we had observed data for specific ages only (0–1-5–10-12–14-16–21-23y). We used mathematical modelling that allows prospective evaluation of each participant in the same time frame and, thus, allows more accurate analysis of BMI data as well as the role of age of onset and length of obesity in the development of cardiometabolic risk later in life. Particularly, we used cubic polynomials to interpolate BMI not measured during assessments [36]. A description of this interpolation method can be found in Correa et al. [36] Spline curves can also be used to forecast or extrapolate values of future time periods beyond the time period of available data. Extrapolation of BMI for short- (less than 5y) and long-term forecasts (more than 5y) is considered in this study. Once the differential between biological and chronological age (which we will estimate with the epigenetic clock) in never-OB, recent-onset-OB, and persistent-OB participants and the correlation with BMI in each group are known, we might be able to forecast their biological age up to the middle-age, based on predicted BMI values. This will be a valuable tool for predicting future health events and healthcare needs in at-risk groups.

#### Structure and composition of the intestinal microbiome

Our raw data will be curated and analyzed using the DADA2 pipeline. In summary, all sequences are filtered to truncate the paired reads to 150nt and eliminate reads with quality values < 2. Taxonomy in samples is assigned using the SILVA database. The resulting amplicon sequence variants (ASVs) are then analyzed using the Phyloseq package in R, and chimeric sequences will be removed using the ChimeraSlayer algorithm. The number of species present in a sample (richness) will be determined using the Chao1 index for alpha diversity. It will be coupled to the abundance of the species (evenness) to calculate overall alpha diversity using Shannon’s index. To gain insights into the intestinal microbiota structure, we will estimate the compositional similarities between microbial communities (beta diversity) using unweighted and weighted UniFrac metrics and the Bray–Curtis dissimilarity analysis. We will use the resulting distance matrix to generate a principal-coordinate ordination plot. To further identify specific taxa contributing to the differences observed amongst samples we will employ correlation analyses on the total community composition via analysis of similarity (ANOSIM) in R and permutation testing of multivariate homogeneity of group dispersions (PERMDISP).

## Discussion

Aging is a natural phenomenon characterized by the accumulation of degenerative processes associated to multiple alterations and damage within molecular pathways. As such, it is the most significant risk factor for all non-communicable diseases (NCDs). Another important contributing factor to the rise in NCDs is obesity. It has been suggested that obesity not only accelerates the onset of metabolic imbalances but also decreases lifespan and impacts cellular and molecular processes in a manner similar to aging. Obesity might accelerate the pace of biological aging, affecting all aspects of physiology and, thus, shortening healthspan and lifespan.

The understanding of aging has increased notably, with current knowledge emphasizing the relevance of ‘network’ theories [47]. Based on these integrative views, aging is best described as a process involving complex interactions between environmental, biological, and molecular mechanisms [13, 14, 47]. The setting for this proposal is a historical cohort of socially vulnerable adults in their early thirties followed since birth (n ~ 1,000). Through the years, we have collected high-quality biospecimens and epidemiological data on nutrition, growth, development, and more recently, cardiometabolic risk aiming to explore the biopsychosocial determinants of obesity and its comorbidities. We have extensive data on socioeconomic and educational status, emotional and behavioral functioning, and personal and family medical histories [9, 34, 36–38]. This is one of the most comprehensive studies of early life risk for developing obesity and related diseases, containing a broad array of environmental, psychological, and biological health markers that we expect to complement with cellular and molecular aging markers. We aim to approach aging as a process whose expression involves the convergence of multiple factors from the early stages of a person's life; understand how longitudinal changes in health trajectories impact the biological mechanisms of aging; and identify potential resilience mechanisms that help prevent unhealthy aging. Moreover, the ObAGE Study will open an opportunity window for futures studies on the transgenerational effects of obesity-induced accelerated aging by enrolling children born to SLS participants and developing collaborative relationships with other prospective epidemiological cohorts, particularly in the Southern Hemisphere.

An accurate definition of biological aging in humans remains a key challenge for aging research. While chronological aging is the time between the moments we are born and die, biological aging is a much broader concept capturing all the differences in how we age. Reliable biomarkers are much needed to understand the molecular and cellular features of aging and how to slow down or even reverse this process. So far, no single molecule or cellular mechanism has been able to explain the progressive decline that comes with aging [48]. However, several options based on high-throughput evaluation of macromolecules derived from biological tissues or cells provide important information to measure biological aging and estimate deviations from chronological age. At the moment, the gold standard for determining the biological age is based on the analysis of DNA methylation patterns of regulatory regions. The so-called epigenetic clock delivers the most accurate measure of biological aging [49], and might be a suitable alternative for the lack of reliable aging biomarkers. Moreover, the epigenetic clock is sensitive to the most common modifiers that alter aging trajectories, such as obesity, exercise, and nutritional interventions [50, 51].

We will use biological samples from the Santiago Longitudinal Study to evaluate the changes in the epigenetic clock for the first time in a Chilean cohort. We will complement such strategy with assessments of a selection of proteins that are currently accepted as age-derived factors associated with nutrient sensing (IGF-1, IGF2), metabolic mediators (Fgf21, Gdf11, Gdf15), cardiac function (Apelin), inflammation (IL-2, IL-6, IL-10, TNFa, SAP), and integrity and health status of the osteo-muscular system (OSM, myostatin, irisin, musclin, and sparc). Funding constraints preclude us from using an experimental approach based on aptamers and mass spectroscopy. However, the palette of proteins selected for our study might be enough to gain deep insight into the molecular aspects of aging in the cohort. Assessment of multiple biomarkers has proven to be a surrogate marker of how humans age in human longitudinal cohorts [52]. Furthermore, combining the epigenetic clock with a subset of biomarkers linked to the hallmarks of aging had been shown to predict lifespan and healthspan assessment [53] effectively.

By 2035, a significant increase in people > 65y is expected in Chile, up to 19% [54]. While a longer life represents important opportunities for older people and society, the extent of these opportunities depends significantly on one factor: health. Although some older people's health variations reflect their genetics [55], most are due to their physical and social environments [56]. Notably, these factors influence aging from childhood [57]. Because social vulnerability usually coexists with exposure to unhealthy foods, lack of exercise, and adverse psychosocial environments [58, 59], people from disadvantaged backgrounds are less likely to add healthy years to their lifespans. While 8% of the Chilean population live in income poverty, 20% live in multidimensional poverty, which entails deprivation in terms of health or healthy living, education, and standard of living [60]; hence the need to approach the social hallmarks of aging along with the biological ones. Likewise, in a country where three in four adults and one in two children have some degree of overnutrition, the challenge of adding healthy years to human lives should also focus on those at risk of obesity-induced accelerated aging, regardless of their age. Healthy aging spans the entire life course and is relevant to everyone, not just those currently in the older age.

The ObAGE Study is a unique possibility to identify systemic, cellular, and molecular biomarkers of obesity-induced accelerated aging and to better understand how obesity may affect the dynamics of aging. Although a single epidemiologic cohort study in a single city (Santiago, Chile) will not be able to answer all questions related to obesity-induced accelerated aging, it will provide an important basis for understanding the connection of obesity with aging. Also, it might serve to encourage similar studies in other countries. Making healthy aging a reality for everyone urgently requires enhancing our knowledge of how exposure to obesity at crucial developmental stages speeds up the aging process from cells to systems, risking the long-term health and wellbeing of a substantial majority.

## Data Availability

Not applicable.

## Abbreviations

BMI: Body-mass index
CRP: C reactive protein
DXA scan: Dual-energy X-ray absorptiometry
FGF21: Fibroblast growth factor 21
GDF11: Growth differentiation factor 11
GDF15: Growth differentiation factor 15
HPA Axis: Hypothalamus–pituitary–adrenocortical
IGF-1: Insulin-like growth factor 1
IGF-2: Insulin-like growth factor 2
IL-2: Interleukin-2
IL-6: Interleukin-6
IL-10: Interleukin-10
IR: Insulin resistance
LC3I: Microtubule-associated light chain 3 I
LC3II: Microtubule-associated light chain 3 II
MECC: Multiple-events case control
NAFLD: Non-alchoholic fatty liver disease
NCD: Non-communicable diseases
ObAGE: Obesity-induced Acclerated Aging
OSM: Oncostatin M
PBMC: Peripheral blood mononuclear cell
p62: Autophagic receptors p62/SQSTM1/A170/ZIP
SAP: Serum amyloid A protein
SLS: Santiago Longitudinal Study
TNF-alpha: Tumor necrosis factor Alpha
WC: Waist circumference
